# Video-based online interviews for palliative care research: A new normal in COVID-19?

**DOI:** 10.1177/0269216321989571

**Published:** 2021-01-21

**Authors:** Geraldine Foley

**Affiliations:** Discipline of Occupational Therapy, School of Medicine, Trinity College Dublin, Trinity Centre for Health Sciences, Dublin, Ireland

There is no doubt that COVID-19 has significantly disrupted the course of health and clinical research. In some cases, researchers may have had to arrest data collection or re-design studies to accommodate to COVID-19. However, despite the challenges of carrying out research during COVID-19, researchers in palliative care are conducting research^1,2^ including research on palliative care needs in the context of COVID-19.^1,2^ The pandemic has necessitated online participation in studies, particularly for studies involving patients and caregivers.^1,3^

Across research fields, we are now beginning to see online data collection moving beyond the confines of surveys, electronic forums, and chat and instant messaging services. Videoconferencing platforms such as Zoom, Webex, GoToMeeting, Skype and Microsoft Teams are being used by researchers in the context of social distancing measures imposed by the pandemic.^4^ In qualitative research, video-based online interviews are emerging as a substitute to traditional ‘in-person’ interviews as researchers and research participants adapt to the conditions of COVID-19.^5^ Videoconferencing platforms allow for maintaining the face-to-face element of interviewing even when the researcher is not physically proximate to participants.

Online interviews with patients and caregivers have not typically featured in palliative care research before COVID-19.^6^ Indeed, video recording of human interaction for research purposes^7^ was far from routine. In qualitative research, interviews can be time intensive and involve degrees of emotional investment by both participants and researchers.^8,9^ Key ethical considerations for traditional in-person interviewing in palliative care research also apply when using video-call platforms to interview. As for traditional in-person interviews, consent is obtained prior to data collection and information surrounding withdrawal and debriefing is communicated before interviewing. Sensitive questioning on sensitive topics, timely interviewing, and researcher self-disclosure, are of equal importance when conducting interviews through video-call platforms as they are when conducting traditional in-person interviews.

We know that online interviewing can limit participation in research along lines of the ‘digital divide’. A lack of access to necessary ICT equipment and to the internet limits the feasibility of online interviewing and while many people in the Global North (and many parts of the Global South) have such access, it cannot be taken for granted in the case of older or disadvantaged population groups. A lack of IT training for research participants might also be a limitation to using videoconferencing platforms. Online interviewing brings additional considerations in relation to privacy and to confidentiality and security of data.^4^ Moreover, the scope for observation in online interviews even with a video component can be understandably less than the scope for observation in traditional in-person interviews. How video-based online interviewing shapes rapport between the researcher and participant deserves close attention.^10^ However, professional videoconferencing platforms do operate securely^4^ and there is little evidence to firmly indicate that building rapport with participants during video-based online interviews is more challenging than building rapport with participants during traditional in-person interviews. Of note, in-person qualitative interviews have been found to be only modestly superior to video-based online qualitative interviews to generate raw data.^11^ For some participants, video-based online interviews are now arguably more convenient and more accessible than the requirement to be physically present in an in-person interview.^12^

There are potential implications for research in palliative care when conducting video-based online interviews which are not necessarily universal to other fields. It is possible that for some patients and/or caregivers or indeed for some healthcare professionals, communication surrounding death and dying through a face-to-face online medium as opposed to a somewhat more sensory medium of a traditional in-person interview could be challenging. Moreover, a remote interview albeit with video, could limit the interviewer’s ability to assist the interviewee navigate through periods of distress simply because s/he is physically distant from the interviewee. Opportunities for the researcher to observe and decode where applicable the complexities and/or nuances in relations between patients and caregivers in palliative care (which help contextualise participants’ experiences) could be less when conducting interviews remotely when compared to conducting interviews in person. However, the option of maintaining privacy in one’s own surrounds during severe illness could appeal to some participants and enable them to feel in control of their surrounds. Last but by no means least, the question as to when (or how appropriate it is) to conduct a video-based online interview with a person at the end of their life requires due consideration.

The context of COVID-19 is altering how we generate data with participants in qualitative research. However, qualitative methods are by their very nature flexible and evolve in response to context.^13^ We now have an opportunity to identify and learn from the benefits and/or challenges of conducting video-based online interviews with patients and caregivers in palliative care research. Online interviewing through videoconferencing platforms in palliative care research might well become a ‘new normal’ in COVID-19.
